# Sporangium Exposure and Spore Release in the Peruvian Maidenhair Fern (*Adiantum peruvianum*, Pteridaceae)

**DOI:** 10.1371/journal.pone.0138495

**Published:** 2015-10-07

**Authors:** Simon Poppinga, Tobias Haushahn, Markus Warnke, Tom Masselter, Thomas Speck

**Affiliations:** 1 Plant Biomechanics Group, University of Freiburg, Botanic Garden, Faculty of Biology, Freiburg im Breisgau, Germany; 2 Freiburg Materials Research Center (FMF), University of Freiburg, Freiburg im Breisgau, Germany; University of Zurich, SWITZERLAND

## Abstract

We investigated the different processes involved in spore liberation in the polypod fern *Adiantum peruvianum* (Pteridaceae). Sporangia are being produced on the undersides of so-called false indusia, which are situated at the abaxial surface of the pinnule margins, and become exposed by a desiccation-induced movement of these pinnule flaps. The complex folding kinematics and functional morphology of false indusia are being described, and we discuss scenarios of movement initiation and passive hydraulic actuation of these structures. High-speed cinematography allowed for analyses of fast sporangium motion and for tracking ejected spores. Separation and liberation of spores from the sporangia are induced by relaxation of the annulus (the ‘throwing arm’ of the sporangium catapult) and conservation of momentum generated during this process, which leads to sporangium bouncing. The ultra-lightweight spores travel through air with a maximum velocity of ~5 m s^-1^, and a launch acceleration of ~6300g is measured. In some cases, the whole sporangium, or parts of it, together with contained spores break away from the false indusium and are shed as a whole. Also, spores can stick together and form spore clumps. Both findings are discussed in the context of wind dispersal.

## Introduction

Polypod ferns, i.e. members of Polypodiales, comprise about 80% of all extant fern species and constitute an important vascular plant group [1,2]. Each specimen produces millions of airborne spores during its life-time [3–5]. Their dissemination is essential for population maintenance, gene flow (migration to another population), and for colonization of new habitats [6]. Spores are reported to have a high migrational ability and to achieve long distance dispersal even between continents and oceanic islands and to withstand hostile environmental conditions during transport [7–9].

**Fig 1:**
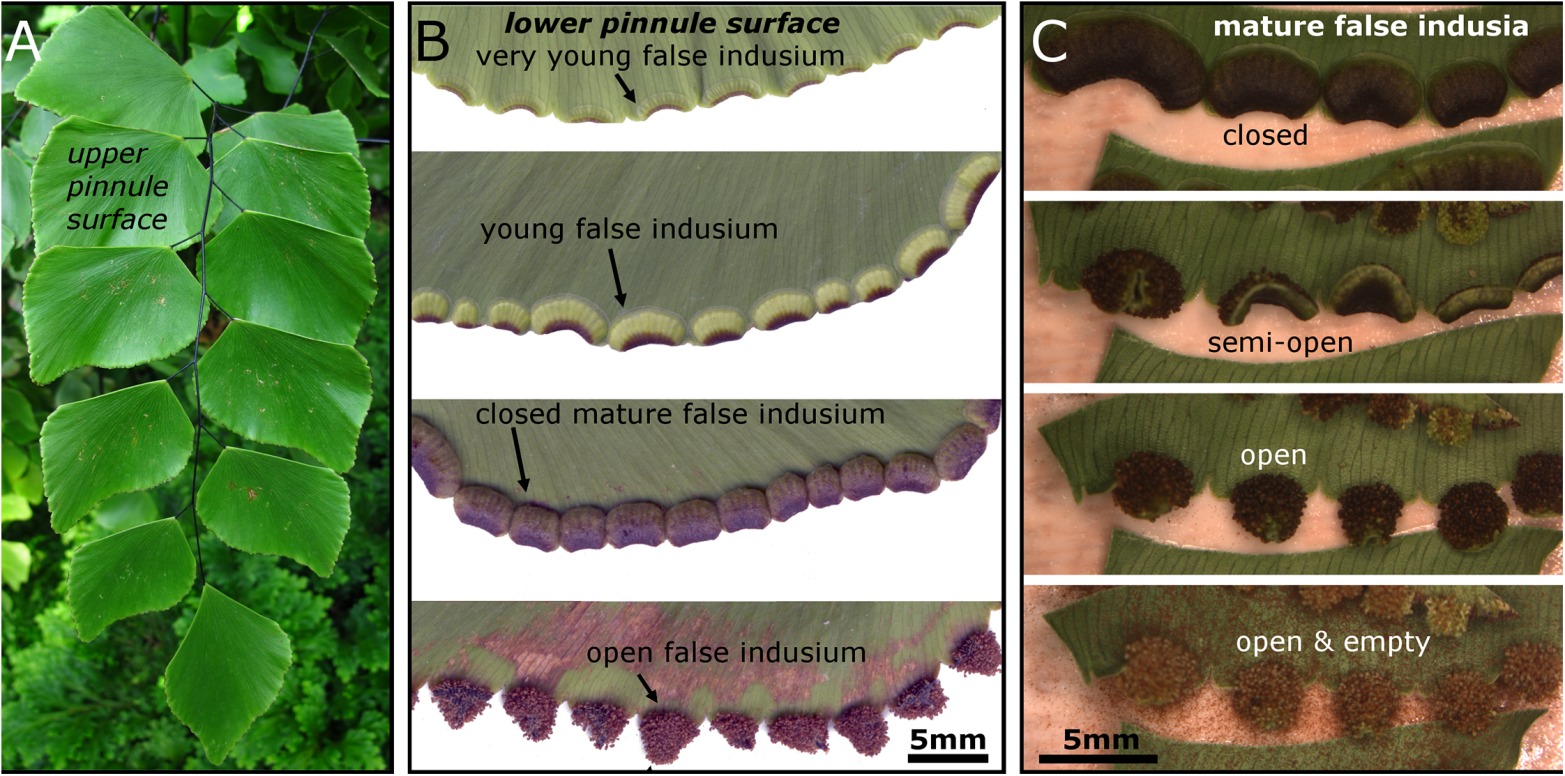
False indusia on *A*. *peruvianum*. (A) A leaflet with several pinnules. (B) The false indusia are being developed on the underside of the pinnule. Several developmental stages of false indusia (note the colors) are shown. The lamina of older pinnules with open false indusia shows signs of degradation (lowermost image). (C) Mature false indusia open completely by desiccation and the exposed sporangia release their spores.

We investigated the spore liberation in cultivated terrestrial *Adiantum peruvianum* Klotzsch (Pteridaceae), commonly known as the Silver-Dollar or Peruvian Maidenhair Fern, which originates from South America (Ecuador to Peru). Naturally, *A*. *peruvianum* grows in shadowy sites of montane rainforests at altitudes up to 1800 m [10,11]. The fronds can reach 1 m in length and 0,8 m in width [12] and are 2-3(4) pinnate. Each of the pinnules features several reniform, strongly curved marginal lobes that remind of kidney basins (Fig 1A and 1B). These so-called false indusia are a few millimeters long and are supplied by several parallel veinlets (cf. [13,14]). True indusia, in contrast, are epidermal outgrowths that do not appear in *A*. *peruvianum*. Concomitant with the development of numerous sporangia on their abaxial surfaces, the false indusia change color from light green to dark brown (Fig 1B) and flap open due to evaporative forcing (cf. [15,16]). The movement of the false indusium leads to sporangium exposure and can be considered as the first step in the process of spore release (Fig 1C).

**Fig 2:**
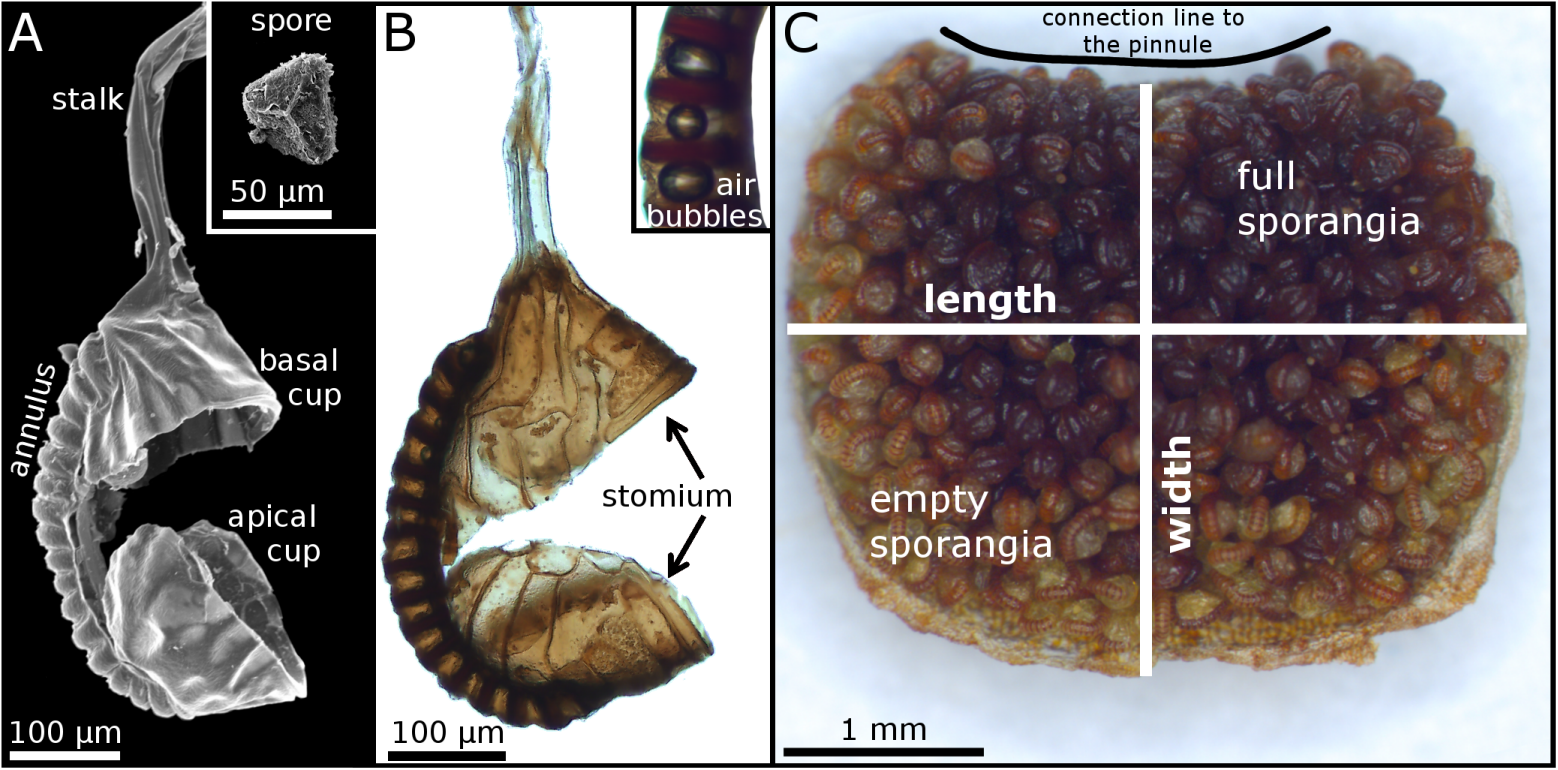
Leptosporangia on the underside of a false indusium of *A*. *peruvianum*. (A) Scanning electron microscopy (SEM) image of a ruptured empty sporangium. The inset shows a trilete spore. (B) Light microscopy (LM) image of a ruptured empty sporangium. The inset shows annular cells with gas bubbles that occurred due to cavitation inside the cells. (C) Underside of a detached false indusium with sporangia that partly have already shed their spores. The connection line to the pinnule margin is indicated.

The second step is the actual spore liberation out of the sporangia. Polypod ferns share a distinct morphological character typical for their mostly globose, a few 100 μm long sporangia: a vertical ring of annular cells which is interrupted by the insertion of the generally thin and long stalk and a lateral stomium region [2,5,17,18] (Fig 2A and 2B). Apart from a few exceptions of indehiscent sporangia [19], the spore liberation after sporangium exposure is enabled by this annulus structure [20–28]. Its cells lose water by evaporation and deform due to an increase of water tension within them, hereby inducing stresses on the leptosporangium as a whole and finally causing tissue rupturing at the stomium region. The ruptured sporangium splits open and two sporangium halves (cups) are formed. As a consequence of the ongoing desiccation process, the annulus uncoils further and draws the apical cup in an arc-shaped motion away from the basal cup which is situated at the base of the sporangium (where the stalk is inserted). The flexible continuous band formed by the lignified thick inner walls of the annulus cells stores elastic energy during the opening process. Upon exceeding the cohesion forces of the water in the annulus cells at about -9 MPa [29], cavitation is caused [30] and gas bubbles form due to cell sap rupture (Fig 2B). The stored elastic energy is converted into kinetic energy: the annulus snaps back, hereby ejecting the spores. Due to the poroelastic properties of the annulus cells the sporangium firstly re-closes only up to 40% of total closure within 10 μs, and this fast movement facilitates the ejection. In other words, the water in the porous annulus cells does not have the time to accelerate and flow during this fast motion, hence water pore pressure increases which finally leads to a stop of the annulus movement. Afterwards, water pore pressure is reduced, water can flow and re-closure proceeds with much slower velocity to ~85% total closure [31].

To our best knowledge, there have not yet been performed detailed analyses on the first step of spore release in *A*. *peruvianum*, i.e, the movement of the false indusium. Moreover, during observations of leptosporangia we noted that spores adhered in about equal parts to both sporangial cups during the process of sporangium dehiscence and that both cups are empty after annulus relaxation. Ingold [27] states (for an unknown species) that the upper cup contains most of the spores during dehiscence and also that both cups are empty afterwards. The catapult functioning of the annulus would mainly affect spores which are situated in the apical cup (the ‘throwing arm’). We investigated the ballistics of the spores and analyzed how the release of kinetic energy during annulus relaxation contributes to the discharge of spores from the basal cup.

## Materials and Methods

### a) Specimen source and general treatments

*A*. *peruvianum* is cultivated in a tropical greenhouse of the Botanical Garden of the University of Freiburg. Freshly cut leaves and pinnules with false indusia were transported and stored in plastic boxes and covered with moist paper tissues to avoid desiccation.

### b) Morphological/anatomical analyses and counting experiments

False indusia and sporangia were analyzed with an Olympus BX61 light microscope (Olympus Corp., Tokyo, Japan) equipped with a DP71 digital camera or with Olympus SZX7 or SZX9 stereo microscopes equipped with an Altra20 or a ColorView II camera. The cell^D 2.6 software (Olympus Corp., Tokyo, Japan) was used. We photographed the undersides of 24 false indusia and measured widths and lengths of the false indusia using the software Fiji / ImageJ [32] (Fig 2C). From 12 photographs the leptosporangia were counted using the Cell Counter plugin for Fiji / ImageJ (written by Kurt De Vos, http://fiji.sc/Cell_Counter). Due to the fact that false indusia are strongly curved and because sporangia are being developed very densely and also overlap each other, these measurements give approximate values of sporangium numbers per false indusium. Only structures that clearly could be identified as a single sporangium (often recognized by the conspicuous annulus) were counted.

False indusia were embedded with Technovit7100 (Heraeus Kulzer GmbH, Wehrheim, Germany) and 5 μm and 10 μm thick semi-thin transverse sections were produced by using a custom-made rotating microtome. All sections were taken at the plane of symmetry of the false indusia. Toluidine-blue staining was applied (infiltration for 1 min in toluidine C.I. 52040, 1 min washing with de-ionized water) and the microscopy slides were sealed with Entellan (Merck KGaA, Darmstadt, Germany).

Scanning electron microscopy imaging of sporangia was conducted with a SEM LEO 435 VP (Leica, Wiesbaden, Germany) and involved the following preparation steps: air drying, mounting on aluminum stubs using conductive double-sided adhesive tabs (Plano GmbH, Wetzlar, Germany) and gold coating (approx. 15 nm) with a Sputter Coater 108 auto (Cressington Scientific Instruments Ltd., Watford, England).

### c) Kinematic analyses

The slow movement of false indusia was recorded on living plants, on cut leaves, on cut pinnules/pinnule margins, or with false indusia which were detached by using a razorblade. If required, the structures were either held with lockable tweezers or were attached to microscopy slides with double-sided adhesive tape (tesa SE, Hamburg, Germany) or with all-purpose adhesive (UHU GmbH & Co. KG, Bühl, Germany). We recorded in greenhouses of the Botanic Garden (living plants) or in our microscopy lab (potted plants, detached leaves / pinnules). Separated false indusia were analyzed in air and submersed in tap water. The cameras used were either a TLC200 Pro timelapse camera (Brinno c/o Phase 3 Systems, Florida, USA) or cameras of the microscope equipment listed for the morphological/anatomical analyses (see above). The recording speed varied between 1 frame per minute and 1 frame per 10 minutes. Temperature and relative air humidity were monitored using the data logger EL-USB-2-LCD+ (Lascar Electronics Inc., Salisbury, UK).

One detached false indusium was recorded during its dehydration motion and simultaneously weighted in a small chamber at ambient temperature of 23° Celsius and approximately 50% air humidity using an UMT2 balance (Mettler-Toledo, Columbus, USA). We determined the mass loss m* of the false indusium (in respect to its mass in the fully hydrated state *m*_we_ and the remaining mass *m*) over time due to water loss (evaporation) according to the formula m* = 100 x (*m*_wet_—*m*) / *m*_wet_. Because of the complex kinematics of the false indusium (see Results & Discussion), a fully satisfying quantification of the motion was not possible.

The fast sporangium movement and the spore ejection were recorded by using an Axioplan light microscope (Carl Zeiss AG, Oberkochen, Germany) or a dissecting microscope Olympus SZX9 with a techno light 270 cold light source (Karl Storz GmbH & Co. KG, Tuttlingen, Germany) or Constellation LEDs (IDT Inc., Tallahassee, USA) and the high-speed camera Motion Pro Y4 with the software Motion Studio 2.08.03 (IDT, Tallahassee, USA). The recording speed varied between 20.000 and 100.000 fps. We used a graticule calibrated to 1 millimeter (Pyser-SGI Ltd., Edenbridge, UK). Motions were recorded in situ (i.e., sporangia were still located on the false indusium) and ex situ (i.e., sporangia were gripped at their stalks with tweezers and then adjusted vertically according to their natural orientation). The temperature during the recordings varied between 22° and 25°Celsius and the relative air humidity between 50–60% (measured with the data logger mentioned above). We recorded six videos which were used to determine the annulus velocity by calculation of the circular arcs that the respective upper cups have travelled over time and six videos for calculation of the velocities of altogether 20 spores. We used the software Motion Studio 2.11.00 (IDT Inc., Tallahassee, USA), Fiji / ImageJ and Excel 2007 (Microsoft, Redmont, USA). For each spore we defined t = 0 ms to correspond to the last frame where no sporangium movement is visible. In 19 of the tracked spores the launch speed could not be calculated because the actual release is not visible in the videos. One spore is an exception because it was situated at the outer margins of the basal cup and is already visible before its ejection. We neglected that some spores slightly fly out of plane during these recordings. For the second and much slower annulus relaxation step we recorded at 1000 fps.

### d) Experiment on the spore dissemination

Eight mature false indusia were each fixed onto the edges of small plastic blocks with all-purpose adhesive (UHU GmbH & Co. KG, Bühl, Germany). The blocks were then each attached onto ~12 cm wide and ~20 cm long, stiff cardboards with double-sided adhesive tape in such a way that the false indusia were orientated perpendicularly to the cardboards. The spore release height varied between ~1 mm and ~5 mm, depending on the point of attachment and on the width of the respective false indusium. The sporangia could release their spores onto the cardboard surfaces which also were covered with double-sided adhesive tape, preventing any structure from rolling after landing. These setups were enclosed wind-protected with plastic caps (~6 cm in height), which also stuck tightly on the adhesive tape, and stored overnight in a climate chamber (~24°Celsius, ~13–23%rh) or in our microscopy lab (~22°-25°Celsius, ~50–60%rh). On the next mornings, each cardboard surface was carefully covered with a transparency and digitized (1200 dpi) with a Scanjet 5590 (Hewlett-Packard, Palo Alto, USA). Due to the more or less varying relevant experimental conditions (mainly rel. humidity and temperature variations of 1°C) and the large size of our test chambers, compared to those used e.g. for fungus spore distance measurements [33], we cannot exclude that free convection air currents occurred during these tests. Therefore, we will not present results on the distances the spores and other structures have traveled.

## Results and Discussion

### a) General morphometric results and an estimation on the spore count per plant

False indusia are 4.4±0.9 mm in length and 2.8±0.2 mm in width (n = 24) and possess 280±83 sporangia (n = 12) on their undersides. Sporangia are 310±17 μm long and 232±17 μm thick (n = 46). The sporangial stalks, each constituted of two cell rows, are 397±35 μm (n = 5) in length and 46±1 μm (n = 4) in thickness. When gently pushing a sporangium, e.g. with tweezers, the stalk easily bends and flips back afterwards due to its elastic properties. The annulus consists of 17–19 cells. We measured an annulus length of 497±23 μm (n = 90) and a thickness of 52±4 μm (n = 7). The trilete spores have a diameter of 50±4 μm (n = 30).

We counted 5–6 small as well as 3–4 large fronds on adult *A*. *peruvianum* plants from our greenhouse. On the small fronds, the number of pinnules varied between 17–34 and on the large fronds between 64–82. The numbers of false indusia on each of these pinnules varied between 21–28, whereas the most apical pinnules developed much fewer (and sometimes no) false indusia. When we consider a hypothetical ‘average’ *A*. *peruvianum* plant with five small and three large fronds, possessing ~300 pinnules with ~6000 false indusia, one would find ~1.600.000 sporangia on this plant, each producing 64 spores (cf. [34]). The total number of spores produced by such a plant would be ~100 Mio, an estimation which is in general accordance with the literature [3–5] and which reflects the immense investment of such a plant in diaspores for wind dispersal.

### b) Initiation of the movement of the false indusium

The opening of false indusia is driven by desiccation (cf. [15,16]). Most presumably, the microclimate inside the cavity formed by the closed false indusium prevents the sporangia from losing water and hence hinders spore ejection before the actual opening of the false indusium. Also true indusia (e.g., in tree ferns) are being considered to serve (in addition to mechanical protection also) for retardation of water loss [35]. Once a mature false indusium becomes detached from its pinnule, the sporangia will begin to eject their spores (Fig 2C).

**Fig 3:**
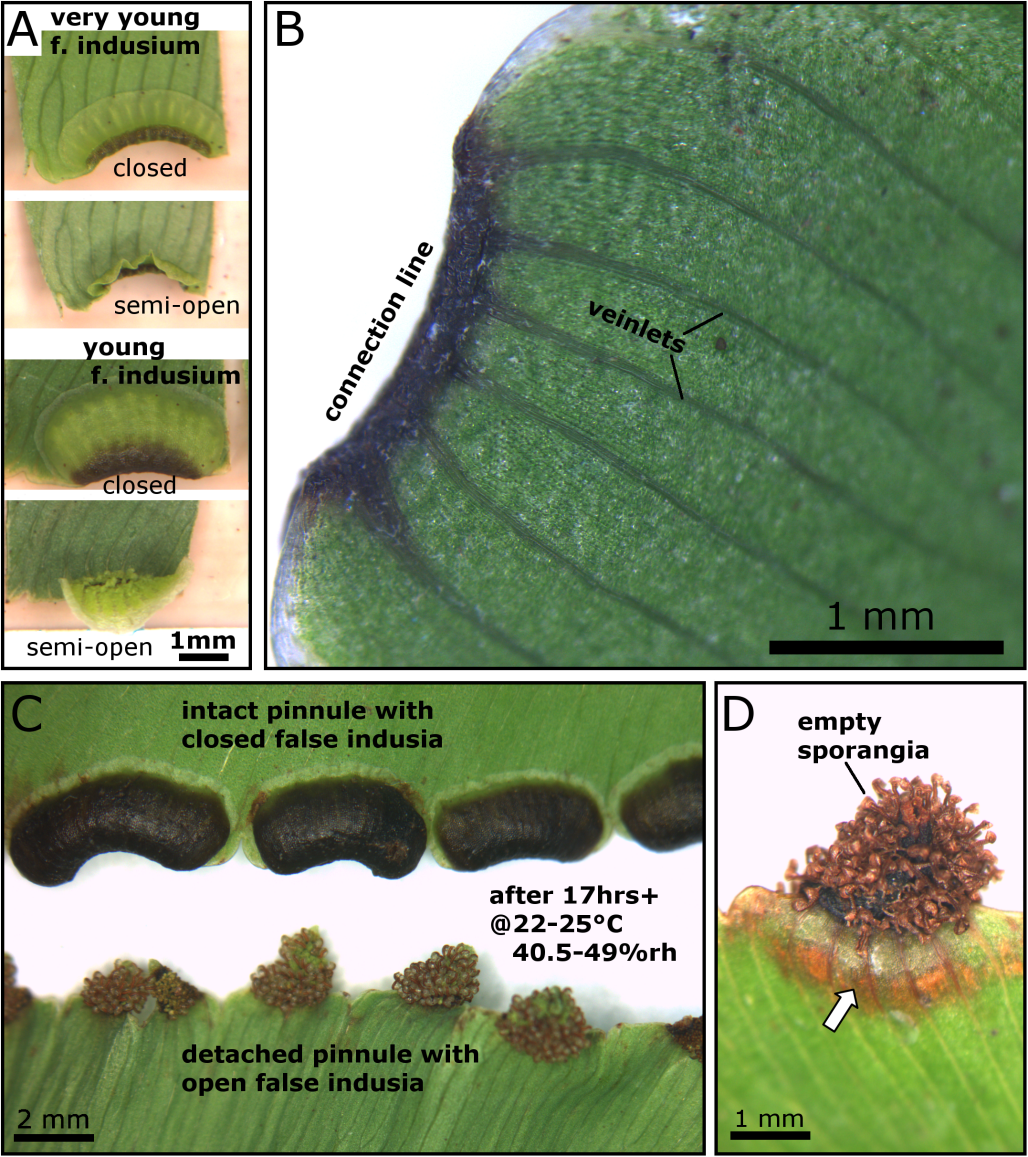
Non-mature and mature false indusia and supplying pinnule veinlets. (A) When non-mature false indusia desiccate (by cutting off the respective pinnule margins), they do not open completely but remain in a semi-open position. No spores become shed (see also S1 Video). (B) View on the adaxial, marginal surface of a pinnule. The connection line between a false indusium, which is situated underneath the lamina, and the pinnule is indicated. The veinlets supplying the false indusium are clearly visible. (C) Details of some of the open and of some of the still closed false indusia after the desiccation experiment described in paragraph 3b). (D) Pinnules with open false indusia show signs of desiccation and degradation in the vicinity of the false indusia (indicated by the arrow).

By cutting off the respective pinnule margins, the desiccation and desiccation-driven movement of immature false indusia can be enforced. They do not open completely and this semi-opening is not accompanied by sporangia opening and a release of spores (Fig 3A) (S1 Video). Such immature structures are being supplied with water from the plant through several veinlets (Fig 3B), which most likely also prevent premature desiccation. Once the false indusia are in a mature stage, the water supply must break down at the ‘right’ time to allow for desiccation and spore release upon favorable environmental conditions. We tested if dry environmental conditions alone trigger the spore shedding processes by simultaneously recording mature false indusia situated 1) on intact pinnules connected to a leaf of a potted healthy and well-watered plant and 2) on detached pinnules from the same leaf. We recorded in our lab for 1065 min (more than 17 hrs) at 22–25°C and 40.5–49%rh. After 8 hrs all of the 78 false indusia from the detached pinnules were completely open. In contrast, even after more than 17 hrs, the 54 false indusia from the intact pinnules (plus roughly several hundred situated on other leaves) were still closed (Fig 3C). In this experiment, the low relative air humidity did not trigger the opening of false indusia on intact pinnules, which indicates that they were still supplied with enough water to prevent the desiccation-driven opening. Older pinnules with open mature false indusia often show signs of degradation (Fig 1B) (cf. [16]) and pinnules with freshly open false indusia show these signs in the vicinity of the connection zone to the false indusia (Fig 3D). We hypothesize that this may reflect a breakdown of the water supply of the pinnule through the veinlets. This finding allows suggesting that a physiologically controlled dieback of this area naturally launches the cascade of the otherwise completely passive, desiccation-driven movements of false indusia and sporangia. Such an explanation is supported by observations in the greenhouses of the Botanic Garden, where mature false indusia on healthy living plants stay in their closed state for several days up to weeks without noticeable motion until they fully open within one day. A detailed investigation on how the motion of the false indusium is initiated by cutting off the water supply and which endogenous and/or exogenous signals naturally trigger this procedure will lead to a more profound understanding of dispersal biology in *A*. *peruvianum* and other polypod ferns.

### c) The kinematics and functional morphology of false indusia

**Fig 4:**
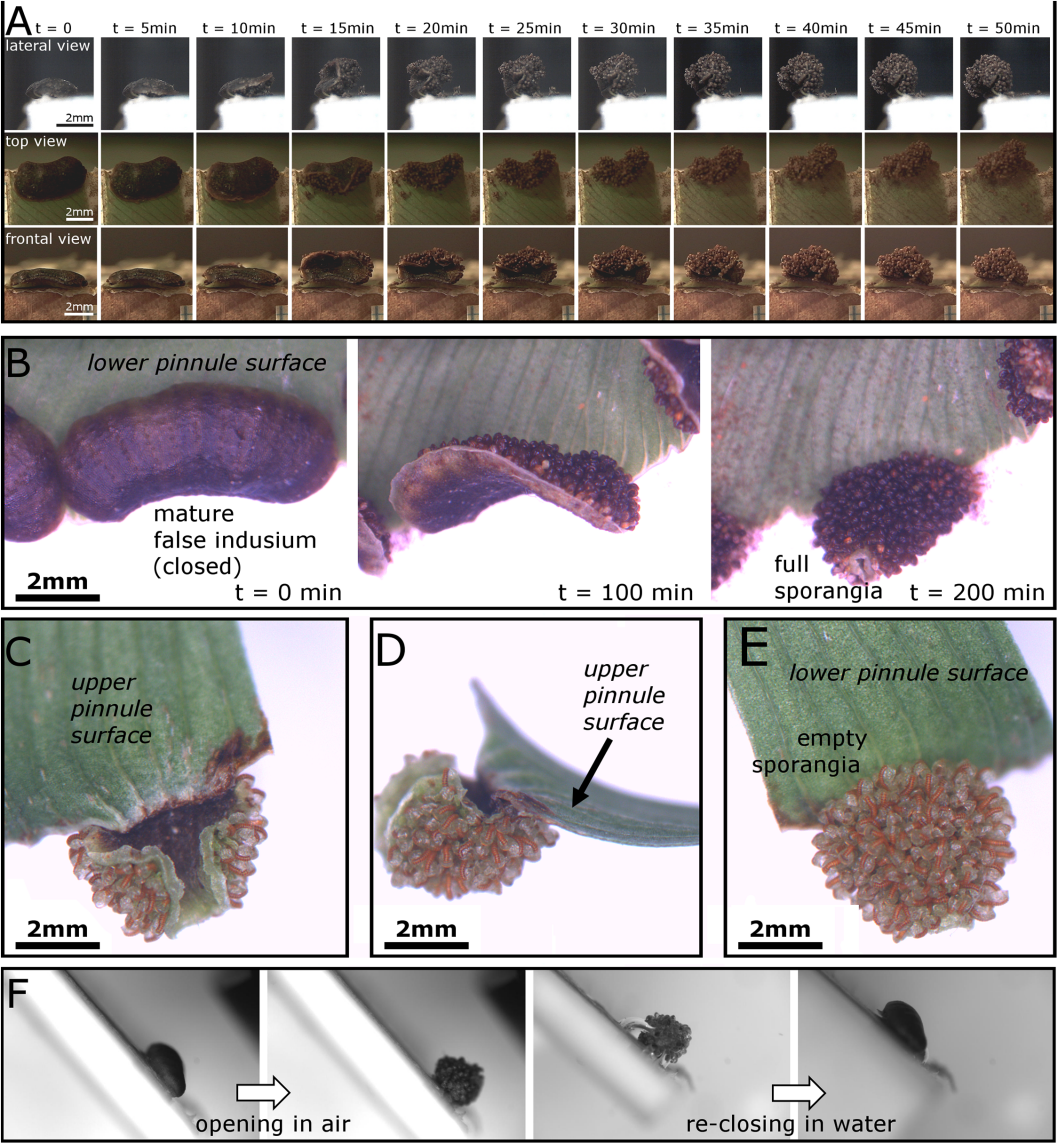
Kinematics of false indusia situated on cut-off pinnule margins. (A) Motion of a false indusium recorded from lateral, frontal and top view in our microscopy lab (~23° Celsius, ~50%rh). The false indusium performs a curvature inversion, rotates around its connection line to the pinnule, and its lateral regions flap towards the middle part. (B) Motion of another false indusium in greater detail. (C-E) When the false indusium is in its fully open state (as seen here from different angles), the sporangia (here empty already) point into different directions, an arrangement for which we hypothesize that it promotes spore scattering. (F) The desiccation-driven opening motion is reversible because open false indusia completely re-close under water.

The findings described and discussed in this paragraph refer to mature false indusia which show a complete opening sequence that is always followed by spore release (Fig 1C) (S1 and S2 Videos). During desiccation, a mature false indusium performs a process of curvature inversion from convex (as seen when recording the adaxial surface) to concave, and the whole structure literally turns inside out (Fig 4A). Concomitant with this continuous shape change, the false indusium rotates around its connection line to the pinnule. In many cases, both its lateral regions flap towards the middle part (Fig 4B). This motion sequence leads to a barrel-shaped form of the dried-out false indusium and causes sporangia exposure in such a way that 1) all sporangia become orientated more or less towards the pinnule margin, and that 2) the sporangia also point in almost 360 degrees around the rotational axis of the ‘barrel’ with the exception of a small gap towards the upper pinnule (Fig 4C–4E). We hypothesize that this complex shape-change promotes effective spore scattering for wind dispersal.

**Fig 5:**
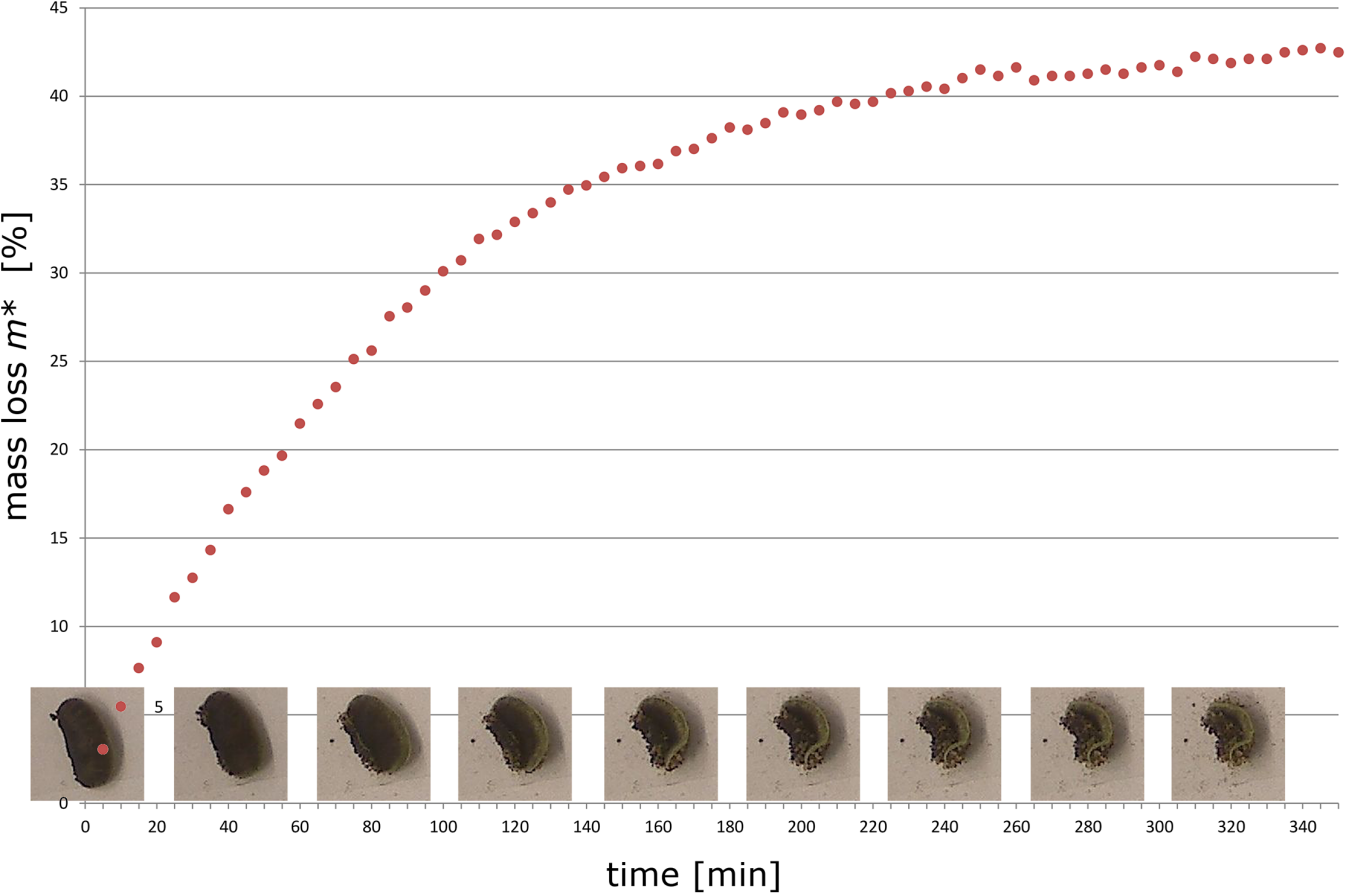
Loss of mass during the movement of a detached false indusium. The movement of the false indusium could not be quantified and is, therefore, qualitatively depicted by presenting single frames in 40 min time steps (the respective S3 Video is recorded with a speed of 1 frame per 5 minutes). The motion is continuous and accompanied by an asymptotic mass loss of up to ~42%.

When transferring open false indusia into water they smoothly reset to their closed condition in a reversed motion pattern (Fig 4F), which shows that the dehydration motion is reversible and is hence not accompanied by substantial structural changes (e.g., tissue rupturing) that would impede the (smooth) re-closing. By continuously filming one detached false indusium in air and weighing it during its movement we observed that the continuous motion starts immediately (S3 Video) and is accompanied by an asymptotic decline of mass (mainly) caused by the loss of water (mass of the false indusium before start of motion: 8.24 μg), with a total mass loss m* of ~42% when the movement is completed (Fig 5).

The durations of false indusia movements were markedly different in our experiments and varied between 50 min (Fig 4A), 200 min (Fig 4B), 350 min (Fig 5) and up to 8 hrs (see paragraph 3b). Theoretically, differences in the dimensions of the false indusia may (partly) explain this observation because the speed of hydraulically actuated plant motion strongly depends on the thickness of the involved tissue(s) [36]. Moreover, our observations indicate that also differences in pinnule remnants, in the environmental conditions (temperature, relative air humidity) and also in the developmental stage of the false indusia influence the durations of the opening sequences.

**Fig 6:**
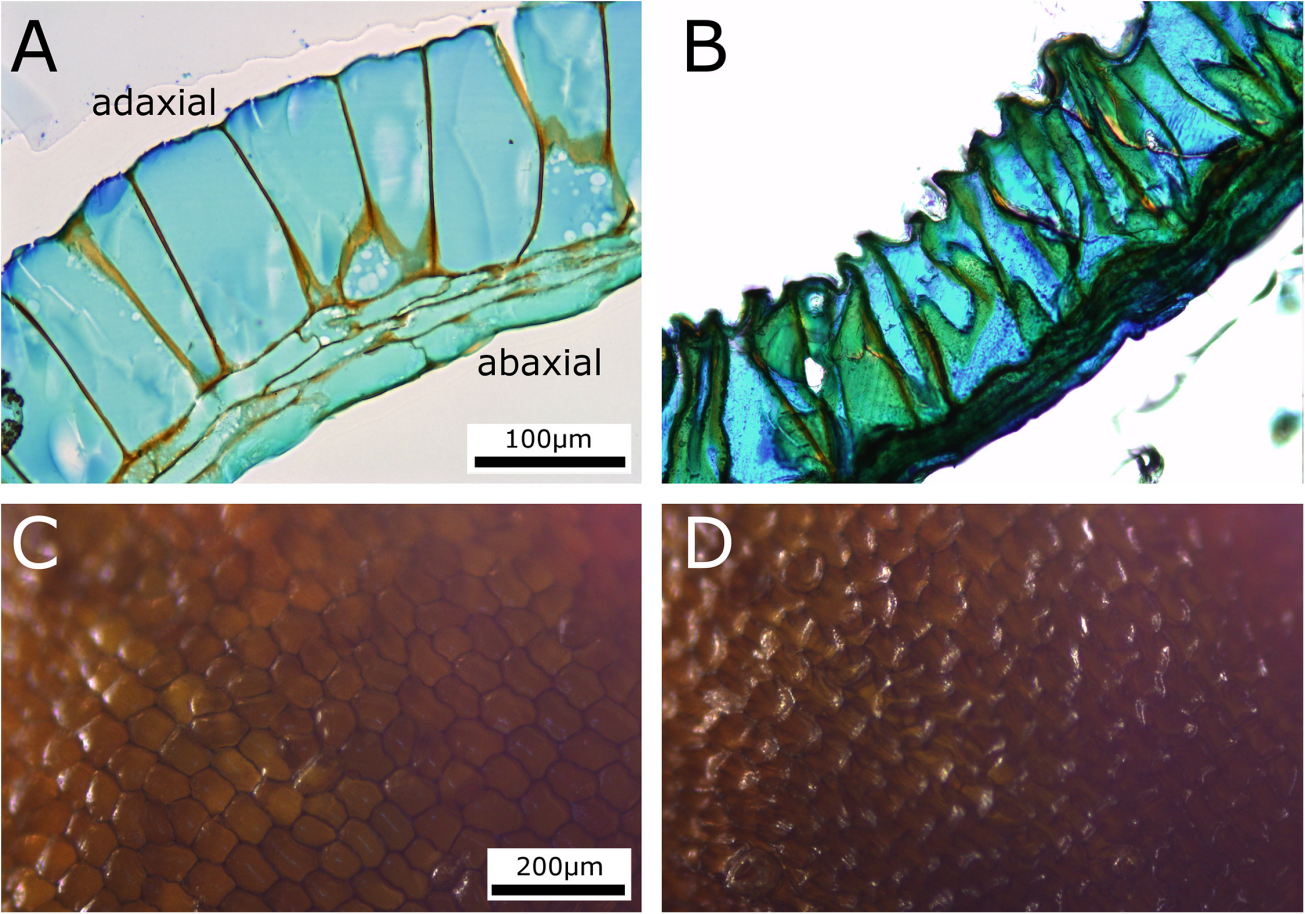
Cellular changes taking place during desiccation of a false indusium. (A-B) LM transverse sections of false indusia in the closed (A) and open (B) state. It is functionally built as a bilayer, with the adaxial layer comprising a single row of large tubular cells and the abaxial functional layer consisting of three rows of tangentially elongated, smaller cells. Especially the adaxial, tubular cells undergo a marked deformation during dehydration. The scale in (A) and (B) is identical. (C-D) The collapse of the adaxial cell walls of the tubular cells (i.e, their curvature inversion towards the cell lumen) is well visible after desiccation (S4 Video). The scale in (C) and (D) is identical.

The false indusium is build up as a functional bilayer, as seen in transverse section (Fig 6A and 6B). Its adaxial layer is constituted of a single row of tubular, upright cells which, when the false indusium is in its closed state (Fig 6A), are of ~150 μm in length and of ~20–80 μm in thickness. The abaxial layer is ~30–60 μm thick and consists of three rows of much thinner, tangentially elongated cells of highly varying lengths. The transverse section of an open false indusium (Fig 6B) reveals that especially the adaxial tubular cells have undergone a conspicuous shrinking process during dehydration. Whereas their overall lengths and the thicknesses at their bases did not change much, their top parts appear constricted (thickness ~20–40 μm) and the adaxial cell walls (which were in contact with air during the motion) are bent towards the cell lumen. During dehydration the thickness of the abaxial functional layer becomes also reduced (~30–50 μm). The deformation of the adaxial cell walls of the tubular cells (i.e, their curvature inversion towards the cell lumen) is also visible when observing the adaxial surface of a false indusium during desiccation (Fig 6C and 6D) (S4 Video).

Obviously, water loss through the adaxial cell walls of the tubular cells entails an increase of water tension inside the cells and deformation of the cell walls. We hypothesize that such a cohesion-force driven deformation of the tubular cells generates enough stress for being the main driving force of the overall movement of the false indusium. Probably, also a desiccation-driven shrinking process of the respective cell walls supports the motion. Passive hydraulic movement actuation of structures for diaspore release can be found among a multitude of plant species, as e.g. summarized in the comprehensive reviews by Elbaum & Abraham and Razghandi et al. [37,38]. In the general scenario for movement actuation proposed by the authors, the adaxial layer constituted of tubular cells contracts, whereas the abaxial layer does not (or, to a lesser degree), which in combination leads to a bending deformation. In contrast to leptosporangia, where a single row of annular cells with modified walls are the structural basis for cohesion-force driven motion [20–28] (a structural setup which is similarly present in anthers of the angiosperm *Rhizinus* [39]), the motion of the false indusium is generated by the laminar arrangement of tubular cells and the existence of a second (resistance) layer. How the complex kinematics of the false indusium are being structurally evoked remains to be investigated. Probably, differences in the cellular arrangement and the mechanical properties of the veinlets, which follow the curvature of the false indusium, dictate the observed complex deformation pattern.

Moreover, the curvature inversion of the false indusium reminds of the trap closure in the carnivorous Venus flytrap (*Dionaea muscipula*). Here, elastic energy is built up and stored in the trap lobes during an active slow hydraulic initial motion which is then released by a passive fast curvature inversion of the lobes (snap-buckling) [40,41]. In contrast, the false indusium shows a smooth transition between the two states of curvature which are obviously not separated by a distinct energy barrier. This allows us to assume that the false indusium shell does not store and release a considerable amount of elastic energy (stretching energy) before or during its motion, respectively. By handling of false indusia we observed that they are quite stiff in their fully hydrated state whereas they feel very soft and flexible when dry. This leads us to hypothesize that such a change of mechanical properties, which is apparently accompanying the dehydration movement, is probably enabling this smooth curvature transition.

### d) Sporangium and spore motion

**Fig 7:**
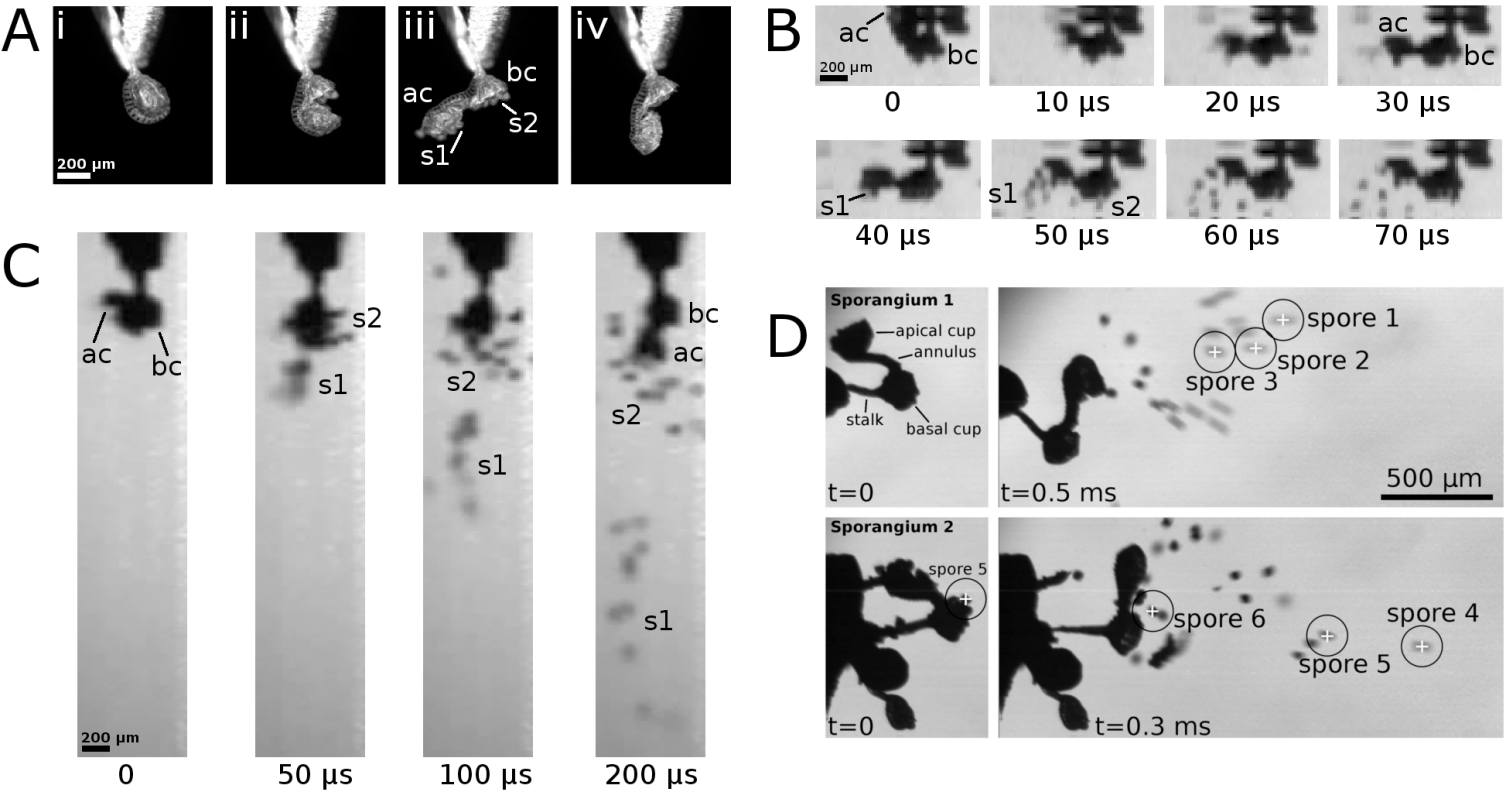
Sporangium motion sequences and spore dissemination. The sporangia have been gripped at their stalks with tweezers and adjusted vertically. Sporangium 2 in (D) is still situated on a false indusium. ac = apical cup; bc = basal cup; s1 = spores from apical cup; s2 = spores from basal cup. (A) The slow passive nastic movement of the annulus includes rupture of the sporangium (i,ii) and formation of the apical cup and basal cup (iii). The annulus relaxes after this tensioning process (including a first ultrafast relaxation step), but does not immediately reset to its initial position, stopping approximately halfway (iv). Afterwards, the annulus further resets; the extent of this step varies and can lead to a nearly initial annulus curvature, or may stop much earlier. (B) First, ultrafast annulus relaxation step which leads to rapid spore ejection from the apical cup. This step lasts only 40 μs until spores emerge in the respective frame. Afterwards the sporangium bounces and spores from the basal cup also become shed (t = 50–70 μs). Frames were taken from S7 Video, cropped and digitally resized without interpolation. (C) The dissemination pattern during spore ejection from a sporangium is shown. At the beginning, the tensioned annulus is turned in such a way that the apical cup points left backwards. After relaxation, spores of the apical cup are forcibly ejected almost straight downwards, whereas those from the basal cup are shed in an irregular pattern. Frames from S11 Video were cropped and digitally resized without interpolation. (D) Tracked spores from S9 and S10 Videos (images rotated 90° anti-clockwise). Still frames of sporangium 1 and 2 at t = 0, which depict the last frames before the respective annuli start to move, and at t = 0.5 ms respective t = 0.3 ms. The six tracked spores are visible and marked. Spore 5 from the basal cup of sporangium 2 is already visible at t = 0.

The sporangia start to move as soon as they have become exposed by deformation of the respective false indusium (Fig 2C) (S1 and S2 Videos). At the beginning of the sporangium movement, the tensile stresses generated by cohesion forces within the annulus cells cause a rupturing of the stomium region, the predetermined breaking region which consists of elongated and lignified cells [20,24,25]. As a consequence, curved cracks emerge at the stomium region and run along both lateral leptosporangium walls towards the annulus ring. The upper half and the lower half of the sporangium capsule hence become increasingly separated, two cups are formed and the apical cup is moved away from the basal one (Fig 7A) (S5 Video). During this process, the cracks reach the annulus ring and proceed further along its inner cell walls by which the annulus ring is connected to the sporangium capsule (Figs 2A, 2B and 7A). Thereby the obovate sporangium capsule detaches partly from the annulus and a flexible continuous band in the middle region of the annulus ring is formed. In some cases a portion of the capsule remains on the middle part of the annulus (S6 Video). The whole, air humidity-dependent tensioning process was measured in our microscopy lab (22°-25°Celsius, 50–60%rh) to last 19±3.2 s (n = 6) (the process shown in S5 Video is much shorter because the initial cracking of the stomium is not shown). During this movement, the spores adhere in approximately equal numbers to both cups (Fig 7A) (S5 Video).

In contrast to former reports [4,28], but also stated by King [24] for a not defined species of Polypodiaceae, spores are not shed during the leptosporangium opening movement, though occasionally single spores may fall out of the cups. It can be speculated that they are held in place by (a combination of): 1) surface tension that links the spores together and to the cups [42]; 2) sticky substances and/or adhesive/complementary structures on the spore wall (cf. [43]); and/or 3) due to electrostatic forces [44–47].

The subsequent annulus relaxation upon cavitation in the annulus cells takes place in two well distinguishable steps. First, the annulus re-snaps to a roughly intermediate position within 40 μs (Fig 7B) (S7 Video) (Noblin *et al*. [31] measured 10 μs for *Polypodium aureum*), hereby reaching a top speed of 6.9 m s^-1^ and a mean speed of 4.0 m s^-1^ (n = 6). Spores from both cups become ejected (S5–S11 Videos). After this step, gas bubbles are visible in the annulus cells (Fig 2B). Secondly, the annulus ring further relaxes in a much slower movement. The extent of this second step varies among the different sporangia, i.e. the movement may stop very early but may also proceed until the annulus has almost reached its initial curvature (cf. [4,31]). In S12 Video the resetting movement lasts approximately 800 ms, but in other cases this process lasted 8–9 s.

The release of kinetic energy during the first fast step of annulus relaxation causes a bouncing of the sporangium as a whole and ejection of isolated spores from both cups. Spores from the apical cup are ejected by the catapult mechanism and those from the basal cup are shaken out. In some cases (Fig 7C) (S11 Video), the expelled spores from the apical and basal cup form two separate, distinct ‘clouds’. In other cases (Fig 7D) (S9 and S10 Videos), the spores are widely scattered and fly in a rather irregular pattern. Among the tracked spores we calculated a mean spore velocity of 2.4±1 m s^-1^ (n = 20), and the fastest spore traveled with 5.0 m s^-1^. Spore 5 (Fig 7D), which is already visible at t = 0 ms on the respective lower cup before its ejection, has a launch speed of 3.1 m s^-1^ (at t = 0.05 ms), which allows to calculate a launch acceleration of 62,000 ms^-2^ (~6300*g*). King [24] calculated spores of leptosporangiate ferns to be thrown out at up to 10 m s^-1^, however, by assuming the impossible perfect conversion of potential to kinetic energy. The successive quick and slow annulus relaxation steps enable for the catapulting of the spores from the apical cup without any structural element to arrest the fast motion [31]. The high annulus velocity during the fast step is needed to propel the ultra-lightweight spores forcibly out of the upper cup so that they can travel against air resistance which tends to quickly decelerate the spores [48,49]. The Reynolds number for fast moving (simplified) spherical spores is ~17 (spore diameter 50 μm, kinematic viscosity of air of 15 x 10^−6^ m^2^ s^-1^, spore velocity 5 m s^-1^).

In all our experiments, the spores of the *A*. *peruvianum* leptosporangia were completely shed after the first recoil motion of the annulus. In contrast to Schneller [4], who investigated other polypod fern species than *A*. *peruvianum*, we did not observe that many spores remain in the cups after the relaxation motion and only become ejected in further motion events (‘repeated spore release’). As Schneller investigated sori (sporangium clusters) which he exposed to consecutive desiccation and wetting processes and performed counts of released spores in each attempt, we assume that different sporangia may successively have ejected their spores during different desiccation processes. As sporangia are densely aggregated (Fig 1C), their reconfiguration to the roughly spherical shape after spore ejection may reduce the risk that the sporangia impede each other’s opening movement in subsequent discharges of different sporangia. It is conceivable that in annuli which stopped their reconfiguration early, cell sap rupture (cavitation) did not take place in all annular cells.

The shaking of both cups, which is a natural consequence of conservation of momentum, leads to the shedding of spores from the basal cup. In many cases also the sporangial stalk bounces, which is very well visible in S8 and S10 Videos (recorded in situ, i.e. with the sporangia still attached to the respective false indusia) and we hypothesize that this also contributes to the dissemination process. Our analyses indicate that the leptosporangia feature two different mechanisms for spore shedding: upon annulus relaxation, the spores of the apical cup are catapulted, and the recoil initiates dispersal of the spores in the basal cup. Approximately half of the spores in the sporangium are ‘actively’ shot whereas the other half becomes ‘passively’ scattered. We interpret the high kinetic energy released with the recoil of the annulus to be necessary to overcome adhesion forces between spores (but see also our reporting of spore clumps in section 3e)) as well as between spores and sporangium walls and thus to be essential for complete ‘active’ and ‘passive’ spore liberation and separation out of the two cups. Conceivably, the spores ejected from the apical cup can rapidly reach turbulent layers of air farther away from the frond surface. The function of the annulus hence is presumably more complex than acting as a mere spore catapult with one throwing arm. We furthermore hypothesize that the mechanisms of catapulting and shaking help to promote wind-dispersal of individual spores as they are likely to become separated by the different flight velocities and the different distances they will travel.

### e) Dissemination pattern

**Fig 8:**
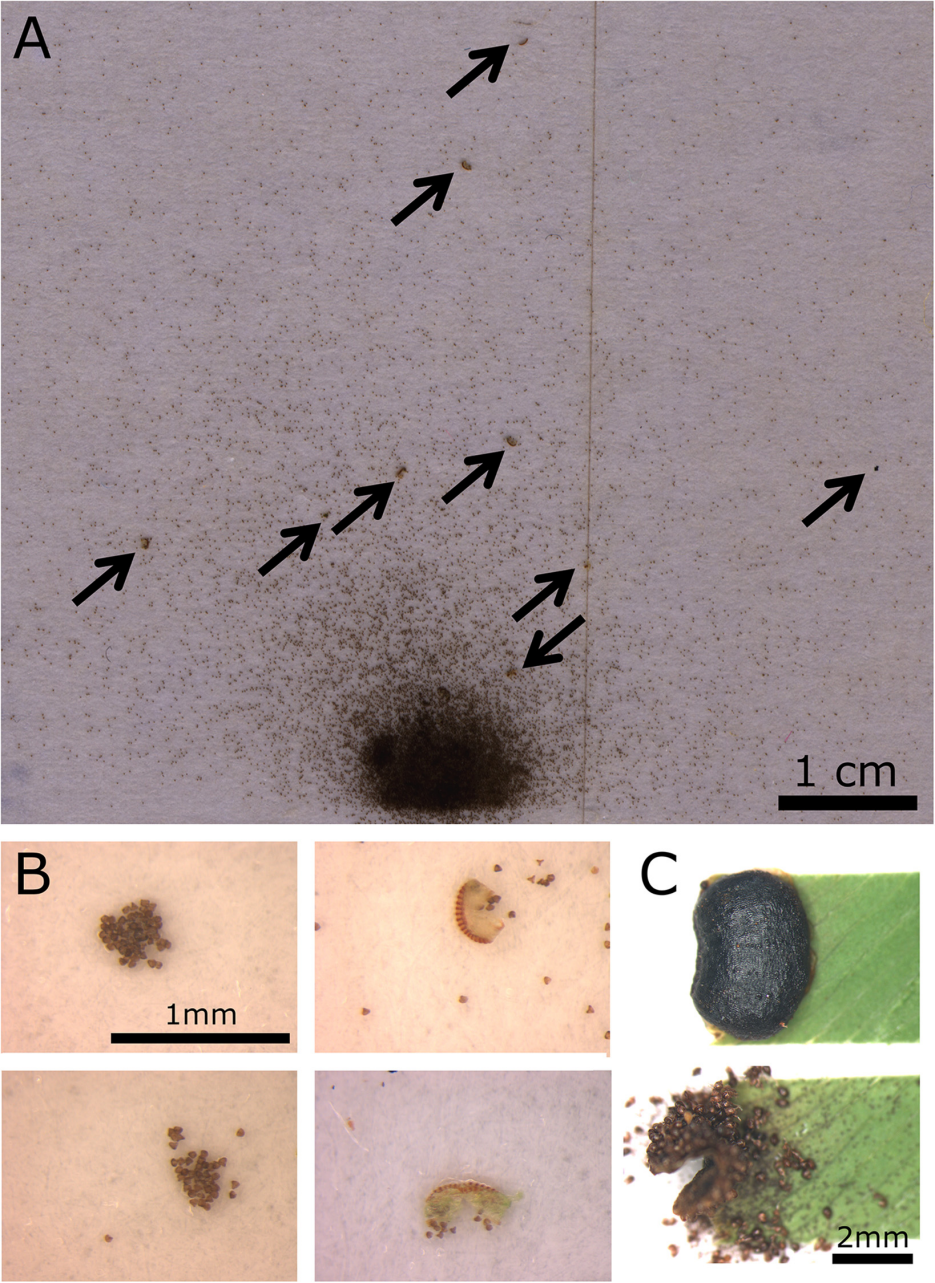
Scattering pattern of spores, sporangia and sporangium fragments. (A) Dissemination pattern of a single false indusium. The arrows indicate sporangial fragments or spore clumps. (B) Left images: Spore clumps observed during the experiments. Right images: Sporangial fragments with spores. (C) During recordings for kinematic analyses we often witnessed the detachment of whole sporangia from the respective false indusium (S13 Video).

Spores from the eight tested false indusia were broadly scattered on the cardboard surface (see exemplary Fig 8A), which is in accordance with our interpretation that the ‘barrel’-form of the open false indusium promotes a spatial separation and dissemination. The spores reached distances (several cm, Fig 8A) that greatly exceed those specified in literature (1–2 cm) [24,27]. This is most presumably caused by air movement due to thermal fluctuations in the test chambers, as indicated in the Materials & Methods section. We observed in all eight experiments that spores may adhere to each other and were ejected as clumps (Fig 8A and 8B). Furthermore, sporangium fragments were often also ejected onto the sticky cardboard surface (Fig 8A and 8B) (cf. 4). In other recordings of false indusia, apart from these dissemination experiments, we also witnessed in some cases that whole sporangia become detached from the false indusium (Fig 8C) (S13 Video). We assume that the release of kinetic energy during sporangium relaxation 1) may cause sporangium rupture or detachment and 2) may not overcome all binding forces between spores which can become ejected as clumps. Spore clumps and spores situated in sporangial fragments (Fig 8B) may have a higher chance to become thrown further distances due to a decreased air friction-induced deceleration indicated by a higher Reynolds number. Scattered whole sporangia may even propel spores from their landing places.

## Conclusion

The four different processes involved in the spore liberation (false indusium opening ~50 min, sporangium dehiscence ~19 s, ultrafast first sporangium relaxation step ~0.00004 s, slower second sporangium relaxation step ~0.8 s) take place in highly different timescales which relate to each other as ~75,000,000 : 475,000 : 1 : 20,000. The opening of false indusia and its final ‘barrel’-like form in the dehydrated state allow for exposure and alignment of sporangia into different directions. The bouncing of the sporangium entails a scattering of spores. During liberation, spores become expelled as individual particles or as spore clumps or are still attached to sporangia or sporangial fragments. As we could show, many different structures influence spore ejection, dispersal and dissemination. This is in contrast to horsetails (*Equisetum* spp.) [50,51] and eusporangiate ferns (*Angiopteris* [52]) where spore liberation and movement is mainly accomplished by the spores themselves via structures of their walls which enable humidity-dependent movements. In addition to becoming shaken or blown out by wind, spores in these plant groups hence can free themselves out of the sporangia which do not forcibly eject them. Both groups share a relative basal position in vascular plant phylogeny [1,5] and in an evolutionary scenario put forward by Hovenkamp *et al*. [52] the advantage of an effective, complete and fast spore ejection and separation led to the evolution of the sophisticated catapult leptosporangium.

The dispersal event following spore release (the distance the spores will travel) is influenced by spore sinking speed, release height, and the occurrence of wind gusts [53]. Spore ejection in more or less downward direction is dictated by the position of the sporangia at the underside of the fern fronds, but this can differ when they are arranged more erect. In other polypod fern species with e.g. erect fertile fronds raised above the sterile ones, ejection most presumably occurs mostly in lateral direction. Many other wind-dispersed plants and fungi are very small and have to shoot their spores into the turbulent boundary layer of air at about 10 cm above ground. Therefore, they need to meet the drag-minimizing requirements for ballistic diaspore travel [54–56]. *A*. *peruvianum* and most other leptosporangiate fern species are relatively tall or exhibit growth forms (e.g. epiphytic) that allow them to drop spores downwards into dispersive air flows. This supports our interpretation that the false indusia and sporangium catapults–with regard to spore dispersal—mainly serve for spore separation and complete ejection rather than to shoot them away as far as possible.

In S8 and S10 Videos the sporangia are still attached to the respective false indusia and the bouncing of the stalks is very well visible. These in situ recordings show sporangia which are standing relatively free and have almost no contact to other sporangia. It remains conceivable that neighboring structures might alter this effect to some extent. Bouncing is also noticeable when the sporangia were recorded ex situ (gripped with tweezers), i.e. when the stalk was artificially shortened (S7 and S9 and S11 Videos). The bouncing depends on the slenderness and elastic properties of the sporangial stalk, as seen here for the long and slender stalks of *A*. *peruvianum*. Sporangial stalks in polypod ferns are generally described as thin and long [5,18]. However, bouncing might also be present in other species with shorter or thicker stalks.

## Supporting Information

S1 File Supplementary Excel file with raw data. (XLS)

S1 Video Time-lapse recording of the dehydration motion of false indusia in different developmental stages. View on detached pinnule margins, recording speed: 1 frame per 5 minutes (MOV, 20 fps, 500 x 371 pixels, 1010 KB).
(MOV)

S2 Video Time-lapse recording of the dehydration motion of mature false indusia. View on a detached pinnule margins, recording speed: 1 frame per minute (MOV, 20 fps, 500 x 371 pixels, 1397 KB).
(MOV)

S3 Video Time-lapse recording of the dehydration motion of a single, detached mature false indusia. Recording speed: 1 frame per 5 minutes (MOV, 20 fps, 130 x 130 pixels, 96 KB).
(MOV)

S4 Video Time-lapse recording of the adaxial surface of a false indusium during dehydration. Recording speed: 2 frames per minute (MOV, 20 fps, 500 x 342 pixels, 386 KB).
(MOV)

S5 Video Slow passive nastic annulus movement and fast relaxation entailing spore ejection. Note that spores adhere to both cups during the tensioning movement and do not yet become shed. The sporangium was gripped with tweezers (real-time MOV, 20 fps, 400 x 200 pixels, 142 KB).
(MOV)

S6 Video Annulus relaxation and spore ejection, recorded with 20.000 fps. The beginning of the slow return (the second relaxation step) of the annulus is well visible. The sporangium was gripped with tweezers (MOV, 20 fps, 832 x 376 pixels, 537 KB).
(MOV)

S7 Video Annulus relaxation and spore ejection, recorded with 100.000 fps. For better visibility, the original movie with a resolution of 140 x 32 pixels was digitally enlarged without interpolation. Furthermore, we added a timescale and chose a very slow playback frame rate in the second part of the video for a better understanding of when the first, very fast annulus relaxation step has ended (at t = 40 μs, the vast majority of spores emerge from the apical cup). The sporangium was gripped with tweezers (MOV, 20 fps, 700 x 160 pixels, 946 KB).
(MOV)

S8 Video Annulus relaxation and spore ejection recorded with 80.000 fps in situ, i.e. the sporangium was still attached to the false indusium. The bouncing of the sporangial stalk is very well visible. For better visibility, the original movie with a resolution of 72 x 110 pixels was digitally enlarged without interpolation (MOV, 20 fps, 432 x 660 pixels, 1750 KB).
(MOV)

S9 Video Annulus relaxation and spore ejection recorded with 20.000 fps. The sporangium was gripped with tweezers (MOV, 20 fps, 176 x 1016 pixels, 807 KB).
(MOV)

S10 Video Annulus relaxation and spore ejection recorded with 20.000 fps. The sporangium was gripped with tweezers (MOV, 20 fps, 176 x 1016 pixels, 707 KB).
(MOV)

S11 Video Annulus relaxation, spore ejection, and spore scattering recorded with 100.000 fps. Note that two distinct spore clouds are formed. The sporangium was gripped with tweezers (MOV, 20 fps, 64 x 800 pixels, 302 KB).
(MOV)

S12 Video Slow return of the annulus towards its almost initial configuration after relaxation. The launch of the spores is not visible due to the comparably slow recording speed of 1000 fps. The sporangium was gripped with tweezers (MOV, 20 fps, 120 x 128 pixels, 642 KB).
(MOV)

S13 Video Time-lapse recording of the dehydration motion of a mature false indusium. Many sporangia detach from the false indusium during the process of spore shedding (view on a detached pinnule margin, recording speed: 1 frame per 5 minutes) (MOV, 20 fps, 500 x 371 pixels, 395 KB).
(MOV)
